# Real-world long-term outcomes based on three therapeutic strategies in very old patients with three-vessel disease

**DOI:** 10.1186/s12872-021-02067-6

**Published:** 2021-06-29

**Authors:** Deshan Yuan, Sida Jia, Ce Zhang, Lin Jiang, Lianjun Xu, Yin Zhang, Jingjing Xu, Ru Liu, Bo Xu, Rutai Hui, Runlin Gao, Zhan Gao, Lei Song, Jinqing Yuan

**Affiliations:** Fu Wai Hospital, National Center for Cardiovascular Diseases, Peking Union Medical College and Chinese Academy of Medical Sciences, No. 167, Beilishi Rd, Xicheng District, Beijing, 100037 China

**Keywords:** Three-vessel disease, Very old patients, Medical therapy, Coronary artery bypass grafting, Percutaneous coronary intervention

## Abstract

**Background:**

There are relatively limited data regarding real-world outcomes in very old patients with three-vessel disease (3VD) receiving different therapeutic strategies. This study aimed to perform analysis of long-term clinical outcomes of medical therapy (MT), coronary artery bypass grafting (CABG), and percutaneous coronary intervention (PCI) in this population.

**Methods:**

We included 711 patients aged ≥ 75 years from a prospective cohort of patients with 3VD. Consecutive enrollment of these patients began from April 2004 to February 2011 at Fu Wai Hospital. Patients were categorized into three groups (MT, n = 296; CABG, n = 129; PCI, n = 286) on the basis of different treatment strategies.

**Results:**

During a median follow-up of 7.25 years, 262 deaths and 354 major adverse cardiac and cerebrovascular events (MACCE) occurred. Multivariate Cox analysis showed that the risk of cardiac death was significantly lower for CABG compared with PCI (adjusted hazard ratio [HR] = 0.475, 95% confidence interval [CI] 0.232–0.974, *P* = 0.042). Additionally, MACCE appeared to show a trend towards a better outcome for CABG (adjusted HR = 0.759, 95% CI 0.536–1.074, *P* = 0.119). Furthermore, CABG was significantly superior in terms of unplanned revascularization (adjusted HR = 0.279, 95% CI 0.079–0.982, *P* = 0.047) and myocardial infarction (adjusted HR = 0.196, 95% CI 0.043–0.892, *P* = 0.035). No significant difference in all-cause death between CABG and PCI was observed. MT had a higher risk of cardiac death than PCI (adjusted HR = 1.636, 95% CI 1.092–2.449, *P* = 0.017). Subgroup analysis showed that there was a significant interaction between treatment strategy (PCI vs. CABG) and sex for MACCE (*P* = 0.026), with a lower risk in men for CABG compared with that of PCI, but not in women.

**Conclusions:**

CABG can be performed with reasonable results in very old patients with 3VD. Sex should be taken into consideration in therapeutic decision-making in this population.

**Supplementary Information:**

The online version contains supplementary material available at 10.1186/s12872-021-02067-6.

## Background

Coronary artery disease (CAD) is common in aging populations [1]. The number of older patients with CAD requiring coronary revascularization is dramatically increasing worldwide [2]. Three-vessel disease (3VD) is a serious type of CAD and accounts for nearly 30% of patients with CAD [3, 4]. Furthermore, 3VD has higher risk of death than that of single-vessel disease [5], especially in older patients who tend to have severe comorbidities. Numerous randomized trials have previously compared the relative results of coronary artery bypass grafting (CABG) versus percutaneous coronary intervention (PCI) in terms of multivessel disease [6–9]. However, these studies did not focus on the very old population with 3VD. Limited real-world data with long-term follow-up are available on outcomes of very old patients with 3VD receiving different treatment strategies. Therefore, the present study aimed to conduct a comprehensive analysis of real-world outcomes among patients with 3VD aged ≥ 75 years undergoing PCI, CABG or medical therapy (MT) alone. The preliminary results of this study were reported in the 30th Great Wall International Congress of Cardiology scientific abstract [10].

## Methods

### Study design and population

This study was derived from an observational cohort consisting of 8943 patients with 3VD. These patients were prospectively and consecutively enrolled at Fu Wai Hospital from April 2004 to February 2011 (Beijing, China). The definition of 3VD was angiographic narrowing of ≥ 50% in the left circumflex, left anterior descending, and right coronary arteries, with or without involvement of the left main artery. In this study, the inclusion criteria were as follows: (1) patients who were ≥ 75 years of age; (2) patients who were willing to be followed up; and (3) patients with complete survival data. The final analysis included 711 patients with 3VD who were eligible. Because we obtained data of this post hoc analysis from a subpopulation of the 3VD cohort, the sample size was determined by the number of patients who met the inclusion criteria instead of being calculated in advance. The choice of therapeutic strategy was based on contemporary clinical guidelines, the judgement of cardiologist teams, and the patient's personal preference [11, 12]. Before the PCI procedure, patients were treated with aspirin and clopidogrel (300 mg or 600 mg, loading dose), and they received standard dual antiplatelet therapy for no less than 1 year after the procedure. The choice of equipment, drugs, and techniques during PCI was at the discretion of the operators. For patients who underwent CABG, standard bypass techniques were used with preferably grafting of the left internal mammary artery to the left anterior descending artery. Experienced surgeons performed the procedure either using on-pump or off-pump surgical techniques on the basis of their individual preference. The ethics committee of Fu Wai Hospital approved this study and it adhered to the Declaration of Helsinki. Informed consent was provided by all participants.

### Study endpoints

We obtained information on the medical history and in-hospital data through the electronic record system of our hospital. Patients were followed up by certificated clinical research coordinators through telephone, follow-up letter, or outpatient visit. The last follow-up was finished on March 2016. All-cause death was the primary endpoint. Major adverse cardiac and cerebrovascular events (MACCE, which consisted of unplanned revascularization, stroke, myocardial infarction and all-cause death) and cardiac death were the secondary endpoints. Unless unequivocal non-cardiac causes could be established, all deaths were considered cardiac deaths.

### Statistical analysis

Data are shown as means with standard deviations to describe continuous variables of baseline characteristics. Percentages and frequencies are shown for categorical variables. Differences in categorical variables of baseline characteristics were compared with the Pearson chi-square test and Fisher's exact test. Differences in continuous variables of baseline characteristics were compared with ANOVA and the Kruskal–Wallis test. A survival curve was shown by using the Kaplan–Meier method and compared by using the log-rank test. Variables that were considered clinically relevant or showed a significant univariate relationship with outcomes were incorporated into multivariate Cox analysis. Variables for adjustment included clinical presentation, age, sex, body mass index, diabetes, hypertension, hyperlipidemia, chronic kidney disease, peripheral artery disease, previous stroke, SYNTAX score, previous myocardial infarction, smoker, left ventricular ejection fraction, and left main disease. Interaction was tested by using the Cox regression model to assess the effects of therapeutic strategies in subgroups. Statistical significance was defined as a two-sided α = 0.05. The statistical analyses mentioned above were performed by using IBM® SPSS® v25.0.0.0 software (IBM Inc., Armonk, NY, USA). Moreover, we performed competing risk regression analysis with sub-distribution hazard models for cardiac death and other cardiovascular endpoints, considering competing risks for non-cardiac death and all-cause death, respectively. Inverse probability of treatment weighting (IPTW) regression analysis based on propensity score was also performed in order to better balance the discrepancies among groups. Standardized mean differences were compared to evaluate the degree of baseline variable balance. A standardized mean difference less than 0.1 was regarded as a high degree of balance. The covariates included in propensity score model were as follows: age, sex, BMI, diabetes, hypertension, hyperlipidemia, previous MI, previous stroke, chronic kidney disease, peripheral artery disease, smoker, clinical presentation, left main disease, LVEF and SYNTAX score. R software (version 4.0.2) was used to perform competing risk and IPTW regression analysis.

## Results

**Fig. 1:**
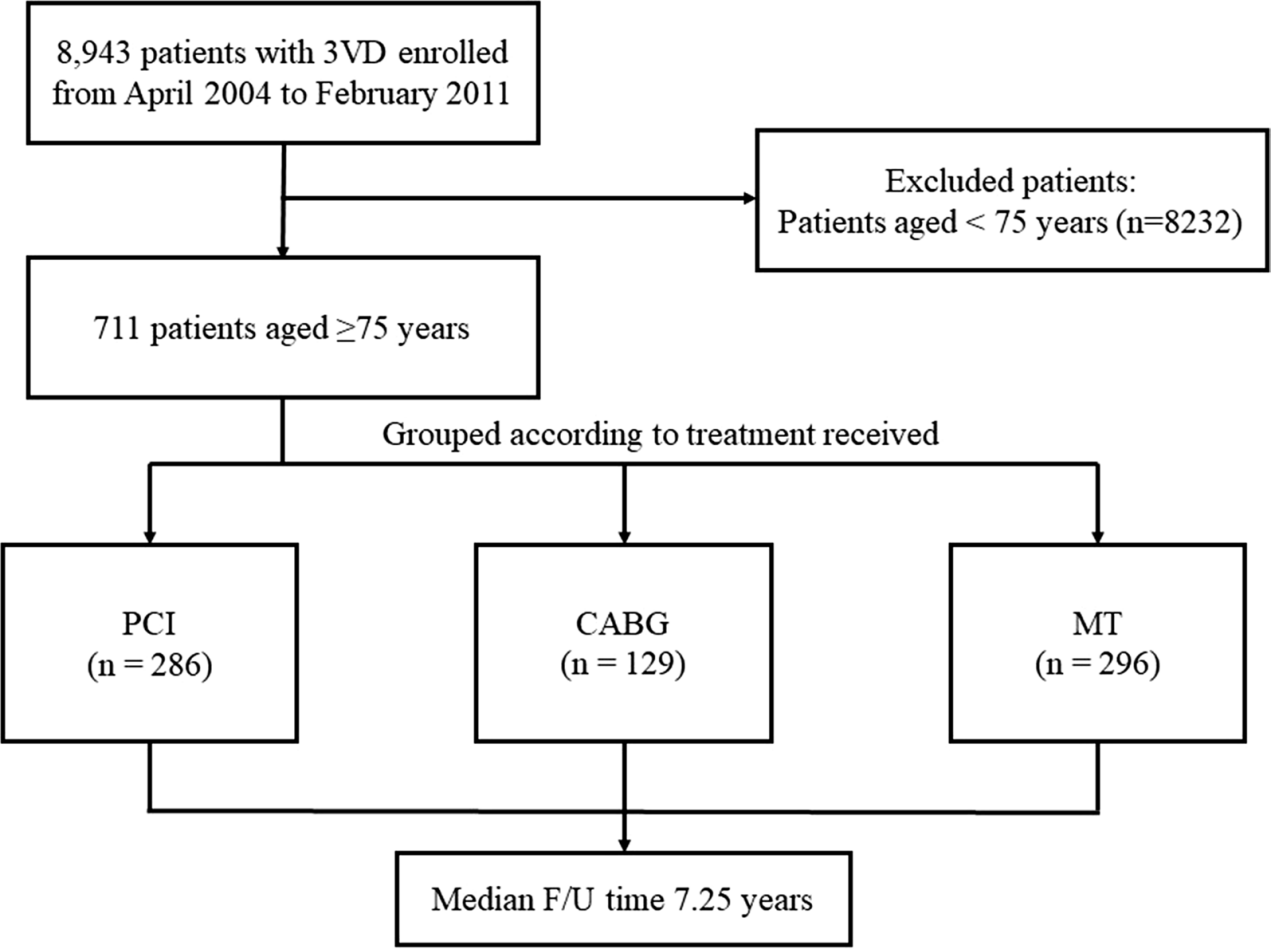
A flow chart for subject selection. *3VD* three-vessel disease, *CABG* coronary artery bypass grafting, *MT* medical therapy, *PCI* percutaneous coronary intervention

This study included 711 patients for analysis, among whom 286 received PCI, 129 received CABG, and 296 received MT (Fig. 1). There were 204 female (28.7%) patients, and the median age was 77 years (interquartile range 75–79 years). Patients in the PCI group were more frequently women compared with those in the other two groups. Patients in the CABG or MT group had higher rates of chronic kidney disease, peripheral artery disease, hyperlipidemia, and significant left main disease, as well as a higher SYNTAX score compared with those in the PCI group (Table 1). The standardized mean difference values of all baseline variables except Clopidogrel after IPTW were less than 0.1 (Additional file 1: Table S1).

**Table 1:** Baseline characteristics of the study population

Variable	PCI (n = 286)	CABG (n = 129)	MT (n = 296)	*P* value
Age (years)	77.4 ± 2.5	76.8 ± 1.9	77.4 ± 2.5	0.029
Female	92 (32.2)	23 (17.8)	89 (30.1)	0.009
Body mass index (kg/m^2^)	25.0 ± 3.0	25.1 ± 3.1	24.9 ± 3.0	0.352
*Risk factors and comorbidities*				
Hypertension	203 (71.0)	93 (72.1)	212 (71.6)	0.970
Diabetes mellitus	103 (36.0)	42 (32.6)	107 (36.1)	0.750
Previous myocardial infarction	85 (29.7)	50 (38.8)	101 (34.1)	0.176
Hyperlipidemia	123 (43.0)	60 (46.5)	162 (54.7)	0.016
Stroke	38 (13.3)	13 (10.1)	42 (14.2)	0.508
Peripheral artery disease	13 (4.5)	23 (17.8)	28 (9.5)	< 0.001
Chronic kidney disease	2 (0.7)	2 (1.6)	11 (3.7)	0.031
Smoker	113 (39.5)	56 (43.4)	123 (41.6)	0.738
*Clinical presentation*				
Stable angina pectoris	81 (28.3)	51 (39.5)	80 (27.0)	0.027
ACS	205 (71.7)	78 (60.5)	216 (73.0)	0.027
Left main disease	57 (19.9)	66 (51.2)	104 (35.1)	< 0.001
Left ventricular ejection fraction < 40%	3 (1.0)	2 (1.6)	5 (1.7)	0.797
Creatinine (μmol/L)	89.7 ± 20.6	89.3 ± 17.8	92.5 ± 26.0	0.251
Creatinine clearance (ml/min)	58.6 ± 14.6	60.4 ± 12.9	57.8 ± 16.3	0.256
*SYNTAX score*				
≤ 22	135 (47.2)	20 (15.5)	76 (25.7)	< 0.001
23–32	103 (36.0)	42 (32.6)	108 (36.5)	0.752
≥ 33	47 (16.4)	67 (51.9)	111 (37.5)	< 0.001
*Medication upon discharge*				
Aspirin	275 (96.2)	119 (92.2)	272 (91.9)	0.082
Clopidogrel	256 (89.5)	11 (8.5)	119 (40.2)	< 0.001
Beta-blockers	242 (84.6)	103 (79.8)	253 (85.5)	0.329
Statins	284 (99.3)	128 (99.2)	295 (99.7)	0.781
Angiotensin converting enzyme inhibitors	277 (96.9)	126 (97.7)	285 (96.3)	0.743
Nitrates	256 (89.5)	117 (90.7)	276 (93.2)	0.271
Calcium channel blockers	278 (97.2)	126 (97.7)	287(97.0)	0.917

*ACS* acute coronary syndrome

**Fig. 2:**
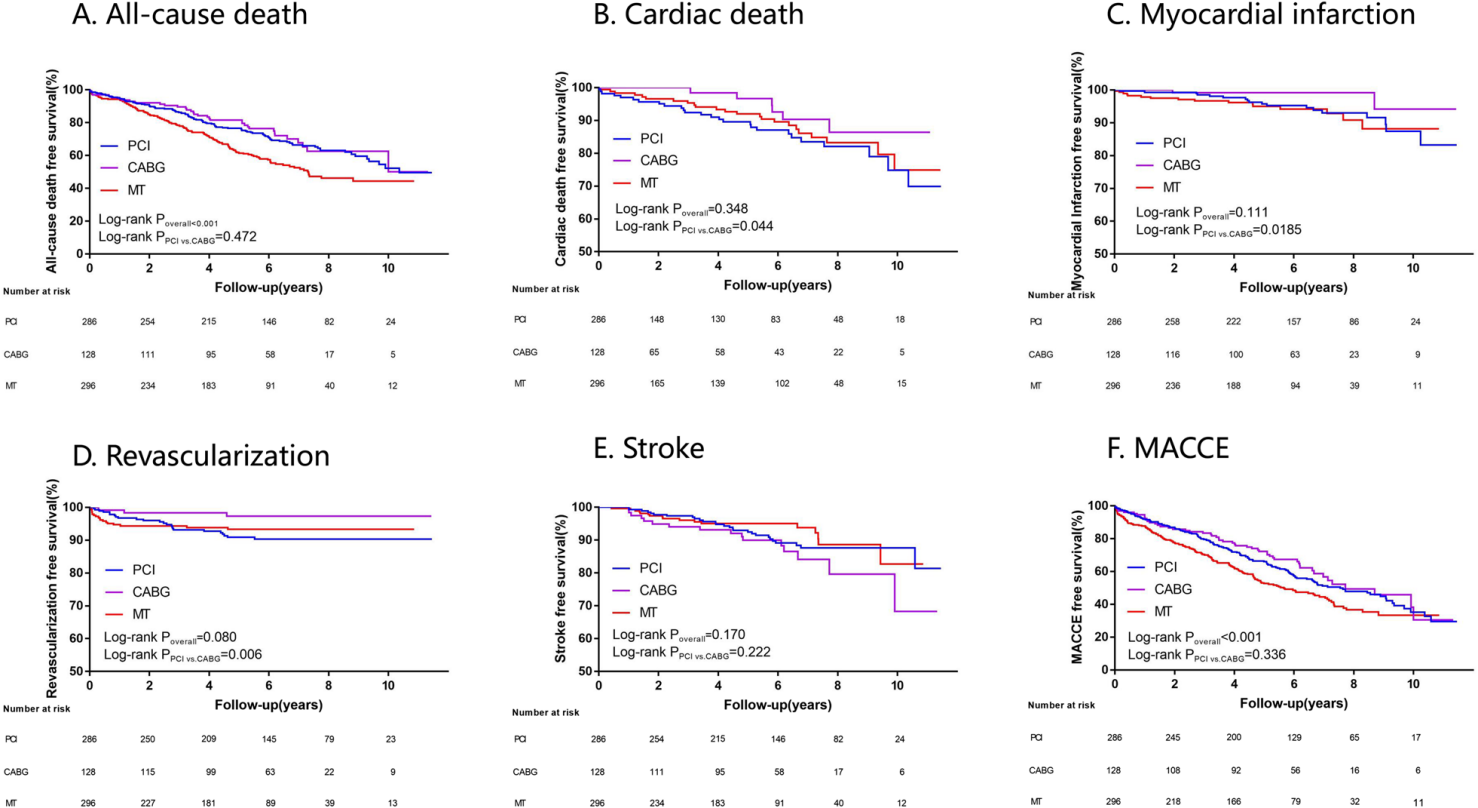
Cumulative survival curves for the primary and secondary endpoints in three treatment groups. Cumulative incidence curves for all-cause death (**a**), cardiac death (**b**), myocardial infarction (**c**), revascularization (**d**), stroke (**e**), and major adverse cardiac and cerebrovascular events (**f**). MACCE: major adverse cardiac and cerebrovascular events

The median follow-up period was 7.25 years (interquartile range 5.75–8.75 years). During this period, 262 patients had all-cause death of whom there were 36 in the CABG group, 99 in the PCI group, and 127 in the MT group. The CABG group showed better results of unplanned revascularization (2.3% vs. 8.4%, *P* = 0.020), myocardial infarction (1.6% vs. 6.3%, *P* = 0.037), and cardiac death (7.8% vs. 15.7%, *P* = 0.026) compared with the PCI group. However, the rates of stroke, MACCE, and all-cause death were not significantly different between these two groups. For patients in the PCI or CABG group, the rates of cardiac death and all-cause death were significantly lower (all *P* < 0.05) compared with those in the MT group. Furthermore, the CABG group had a significantly higher rate of stroke (12.4% vs. 5.7%, *P* = 0.018) and lower rate of MACCE (41.1% vs. 53.0%, *P* = 0.023) compared with the MT group (Table 2). Kaplan–Meier curves for the overall study population showed similar results (Fig. 2).

**Table 2:** Long-term primary and secondary outcomes

Events	Treatment Strategies	No. of Patients with event (%)	*P* value	
All-cause death	PCI (n = 286)	99 (34.6)	PCI versus CABG	0.177
	CABG (n = 129)	36 (27.9)	PCI versus MT	0.040
	MT (n = 296)	127 (42.9)	CABG versus MT	0.003
			*Overall*	*0.008*
Cardiac death	PCI (n = 286)	45 (15.7)	PCI versus CABG	0.026
	CABG (n = 129)	10 (7.8)	PCI versus MT	0.002
	MT (n = 296)	77 (26.0)	CABG versus MT	< 0.001
			*Overall*	< *0.001*
MACCE	PCI (n = 286)	144 (50.3)	PCI versus CABG	0.080
	CABG (n = 129)	53 (41.1)	PCI versus MT	0.516
	MT (n = 296)	157 (53.0)	CABG versus MT	0.023
			*Overall*	*0.074*
Myocardial infarction	PCI (n = 286)	18 (6.3)	PCI versus CABG	0.037
	CABG (n = 129)	2 (1.6)	PCI versus MT	0.648
	MT (n = 296)	16 (5.4)	CABG versus MT	0.070
			*Overall*	*0.117*
Stroke	PCI (n = 286)	27 (9.4)	PCI versus CABG	0.359
	CABG (n = 129)	16 (12.4)	PCI versus MT	0.092
	MT (n = 296)	17 (5.7)	CABG versus MT	0.018
			*Overall*	*0.056*
Unplanned revascularization	PCI (n = 286)	24 (8.4)	PCI versus CABG	0.020
	CABG (n = 129)	3 (2.3)	PCI versus MT	0.282
	MT (n = 296)	18 (6.1)	CABG versus MT	0.100
			*Overall*	*0.062*

Italic values present* P* value < 0.001

Variables that were adjusted for in multivariate analysis included clinical presentation, age, sex, body mass index, diabetes, hypertension, hyperlipidemia, chronic kidney disease, peripheral artery disease, previous stroke, the SYNTAX score, previous myocardial infarction, smoker, left ventricular ejection fraction, and left main disease. Variables that were entered into multivariate Cox analysis were considered clinically relevant or showed a univariate relationship with outcomes. Multivariate analysis showed that the outcomes of unplanned revascularization (adjusted hazard ratio [HR] = 0.279, 95% confidence interval [CI] 0.079–0.982, *P* = 0.047), myocardial infarction (adjusted HR = 0.196, 95% CI 0.043–0.892, P = 0.035), and cardiac death (adjusted HR = 0.475, 95% CI 0.232–0.974, P = 0.042) were significantly better with CABG than with PCI. Furthermore, MACCE appeared to show a trend towards a better outcome for CABG (adjusted HR = 0.759, 95% CI 0.536–1.074, *P* = 0.119). PCI was related to a similar risk of long-term stroke and all-cause death compared with CABG, and was related to a lower risk of cardiac death (adjusted HR = 0.611, 95% CI 0.408–0.915, *P* = 0.017) compared with MT (Table 3). Furthermore, similar results were obtained by competing risk and IPTW regression analysis (Additional file 2: Table S2, Additional file 3: Table S3).

**Table 3:**
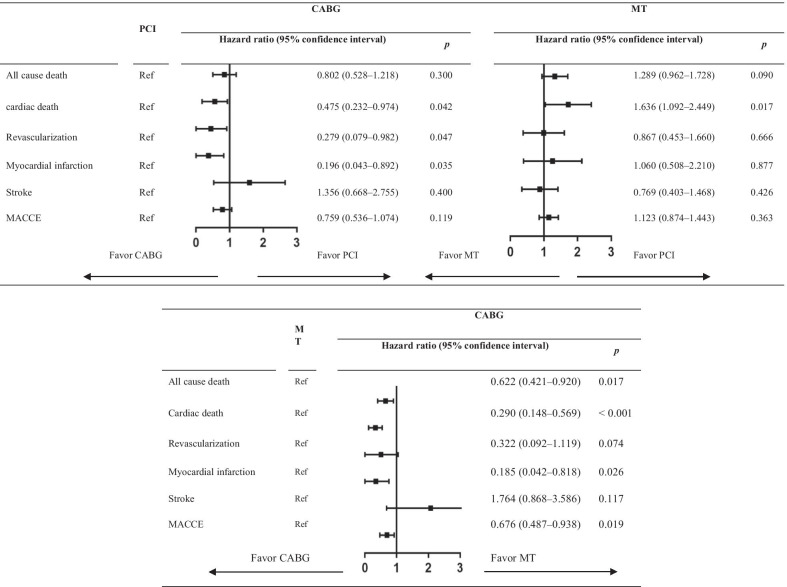
Multivariate cox regression analysis of different treatment strategies on clinical outcomes

*MACCE* major adverse cardiac and cerebrovascular events

Subgroup analysis showed a significant interaction between treatment strategy (PCI vs. CABG) and sex for MACCE (*P* = 0.026). In male patients, CABG showed significantly better results regarding MACCE than PCI (HR = 0.560, 95% CI 0.378–0.831). However, in female patients, these two strategies had a similar benefit of survival from MACCE (HR = 2.064, 95% CI 0.991–4.301) (Table 4).

**Table 4:**
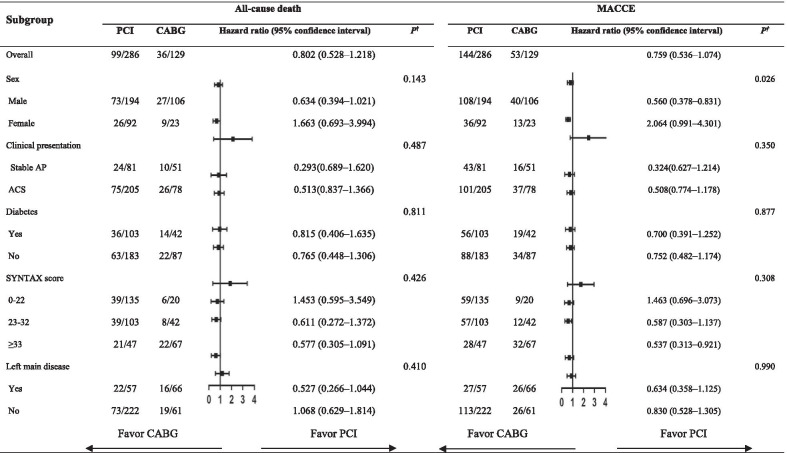
Subgroup analysis on all-cause death and MACCE between PCI and CABG

*MACCE* major adverse cardiac and cerebrovascular events, *Stable AP* stable angina pectoris, *ACS* acute coronary syndrome

^†^ *P* value for interaction in each subgroup analysis

## Discussion

In the present study, our main findings were as follows. (1) CABG was related to lower risk of long-term unplanned revascularization, myocardial infarction, and cardiac death compared with PCI. (2) There was no difference in the rate of all-cause death between CABG and PCI. (3) The interaction effect between sex and treatment strategy (PCI vs. CABG) was significant for MACCE.

In recent years, with the techniques of PCI and CABG dramatically advancing, the relative benefits of CABG versus PCI in patients with multivessel disease have been debated regarding the long-term prognosis [6–9, 13–16]. Generally, CABG tends to have a better benefit regarding all-cause death or MACCE/MACE. Among patients with multivessel disease, a large proportion of these patients are older adults resulting from their increased longevity [17, 18]. However, there have been no randomized trials for comparing the effect of PCI and CABG in very old patients with multivessel disease, such as 3VD. Moreover, results from previous observational studies and pooled-analysis were heterogeneous [19–22]. Therefore, questions persist concerning the optimal treatment strategy of very old patients with 3VD.

We found that CABG significantly reduced long-term cardiac death, as well as unplanned revascularization and myocardial infarction, compared with PCI. Similarly, the SYNTAX trial [23] showed that, for patients with complex CAD, CABG was related to a significant reduction in cardiac mortality versus PCI. This was mainly because CABG reduced myocardial infarction-related mortality, particularly in patients with 3VD. The CREDO Kyoto sub-analysis study on age and sex showed that the interaction effects between age and therapeutic strategies (PCI vs. CABG) was significant for cardiac death. There was also an excess risk of mortality in patients aged ≥ 74 years with 3VD, while there was a neutral risk in younger patients [24]. In real clinical practice, older patients prefer less invasive PCI rather than CABG compared with younger patients. The present study suggested that CABG could also be an advisable option for very old patients aged ≥ 75 years, especially in those who have complex coronary anatomy unfavorable for PCI. This finding might help further decision-making on the choices of coronary revascularization strategies in patients with 3VD.

All-cause death in our study was comparable between the CABG and PCI groups. A previous study reported that CABG was better for an older age compared with PCI in patients with 3VD [24]. However, another study showed that long-term outcomes after CABG tended to be worse in older patients than in younger patients [25]. Older patients have increased risk factors and higher comorbidities than younger patients, as well as a limited life expectancy. These factors may impair the long-term advantages of CABG over PCI in reducing the risk of all-cause death. This is because a substantial number of older patients are likely to die from non-cardiac causes during a relatively long follow-up period. Indeed, we found that cardiac causes only accounted for approximately 40% of deaths in the CABG and PCI groups. The majority of deaths in the CABG and PCI group were non-cardiac, which supports our view to a certain extent. Additionally, the rate of stroke in the CABG group was higher than that in the PCI group, but this was not significant. Older patients are considered to have more perioperative strokes after CABG than after PCI [26]. However, the risk of stroke after CABG has significantly decreased because of increasingly refined perioperative management and operative techniques in recent years [27]. In our study, the risk of all-cause death was not significantly different between MT and PCI. However, PCI significantly reduced the risk of cardiac death compared with MT, which suggested that PCI might have a benefit for the heart.

Notably, we found a significant interaction between treatment strategy (PCI vs. CABG) and sex for MACCE. In male patients, CABG was significantly associated with a decreased risk of MACCE compared with PCI, while no such association was observed in female patients. A prospective multicenter registry showed that male patients with multivessel disease presenting with acute non-ST-segment elevation myocardial infarction had improved survival and reduced MACE with CABG compared with PCI at 5 years [28]. However, in female patients, a long-term benefit from CABG was not observed in this previous study. The difference between sexes could be related to factors such as coronary artery size, the amount of grafts received, use of arterial grafts, and diverse surgical methods [29–31]. Therefore, sex should be taken into consideration in selecting revascularization strategies for patients aged ≥ 75 years with 3VD.

Several limitations of this study need to be addressed. First, although multivariate analysis was performed to evaluate many confounders, residual confounding from other unmeasured factors might have been present. Second, generalization of results might be limited because data were obtained from a single-center cohort and the sample size was relatively small. More studies with a larger sample size are warranted to further confirm our study findings. Third, more specific details about revascularization procedures and drug information as well as functional tests for ischemia would be useful. However, such data were not collected.

## Conclusion

CABG can be performed with reasonable results in very old patients with 3VD. Sex should be taken into consideration in therapeutic decision-making in this population.

## Supplementary Information

**Additional file 1: Table S1**. Baseline characteristics of the study population before and after inverse probability of treatment weighting.

**Additional file 2: Table S2**. Competing risks regression for cardiac death and other cardiovascular endpoints.

**Additional file 3: Table S3**. Inverse probability of treatment weighting (IPTW) regression analysis based on propensity score.

## Data Availability

The datasets analyzed during the current study are available from the corresponding author on reasonable request.

## Abbreviations

3VD: three-vessel disease
CABG: coronary artery bypass grafting
CAD: coronary artery disease
LVEF: left ventricular ejection fraction
MACCE: major adverse cardiac and cerebrovascular events
MACE: major adverse cardiovascular events
MI: myocardial infarction
MT: medical therapy
PCI: percutaneous coronary intervention
