# Supplementary material for: Real-world long-term outcomes based on three therapeutic strategies in very old patients with three-vessel disease
Source: BMC Cardiovasc Disord. 2021 Jun 29;21:316. doi: 10.1186/s12872-021-02067-6 (PMC8243749; doi:10.1186/s12872-021-02067-6)
Supplement: Supplementary file 1 — Additional file 1: Table S1. Baseline characteristics of the study population before and after inverse probability of treatment weighting. [file 12872_2021_2067_MOESM1_ESM.docx]

**Table S1. Baseline characteristics of the study population before and after inverse probability of treatment weighting.**

|  | Not IPTW weighted | | | | IPTW weighted | | | |
| --- | --- | --- | --- | --- | --- | --- | --- | --- |
| Variable | PCI  (n=286) | CABG  (n=129) | MT  (n=296) | SMD | PCI  (n=702) | CABG  (n=734) | MT  (n=716) | SMD |
| Age, yrs **^a^** | 77.42 (2.52) | 76.79 (1.92) | 77.41 (2.47) | 0.187 | 77.31 (2.46) | 77.19 (2.01) | 77.37 (2.45) | 0.054 |
| Female **^a^** | 92 (32.2) | 23 (17.8) | 89 (30.1) | 0.224 | 206 (29.4) | 247 (33.6) | 203 (28.3) | 0.077 |
| Body mass index (kg/m^2^) **^a^** | 25.00 (3.04) | 25.05 (3.06) | 24.68 (2.98) | 0.080 | 24.97 (3.26) | 24.86 (3.16) | 24.87 (3.00) | 0.024 |
| Risk factors and comorbidities |  |  |  |  |  |  |  |  |
| Hypertension **^a^** | 203 (71.0) | 93 (72.1) | 212 (71.6) | 0.016 | 497 (70.8) | 545 (74.3) | 504 (70.4) | 0.058 |
| Diabetes mellitus **^a^** | 103 (36.0) | 42 (32.6) | 107 (36.1) | 0.050 | 252 (35.9) | 310 (42.2) | 252 (35.2) | 0.096 |
| Previous myocardial infarction **^a^** | 85 (29.7) | 50 (38.8) | 101 (34.1) | 0.127 | 249 (35.5) | 246 (33.6) | 237 (33.1) | 0.033 |
| Hyperlipidemia **^a^** | 123 (43.0) | 60 (46.5) | 162 (54.7) | 0.157 | 316 (45.0) | 358 (48.8) | 345 (48.1) | 0.051 |
| Stroke **^a^** | 38 (13.3) | 13 (10.1) | 42 (14.2) | 0.086 | 70 (10.0) | 92 (12.5) | 87 (12.2) | 0.053 |
| Peripheral artery disease**^a^** | 13 (4.5) | 23 (17.8) | 28 (9.5) | 0.290 | 56 (7.9) | 80 (10.9) | 64 (9.0) | 0.068 |
| Chronic kidney disease **^a^** | 2 (0.7) | 2 (1.6) | 11 (3.7) | 0.141 | 20 (2.8) | 10 (1.4) | 15 (2.1) | 0.067 |
| Smoker **^a^** | 113 (39.5) | 56 (43.4) | 123 (41.6) | 0.053 | 297 (42.4) | 338 (46.2) | 299 (41.7) | 0.058 |
| Clinical Presentation **^a^** |  |  |  | 0.178 |  |  |  | 0.057 |
| Stable angina pectoris | 81 (28.3) | 51 (39.5) | 80 (27.0) |  | 219 (31.2) | 201 (27.3) | 213 (29.7) |  |
| ACS | 205 (71.7) | 78 (60.5) | 216 (73.0) |  | 483 (68.8) | 533 (72.7) | 503 (70.3) |  |
| Left main disease **^a^** | 57 (19.9) | 66 (51.2) | 104 (35.1) | 0.455 | 227 (32.3) | 212 (29.0) | 232 (32.4) | 0.050 |
| Left ventricular ejection fraction < 40% **^a^** | 3 (1.0) | 2 (1.6) | 5 (1.7) | 0.161 | 31 (4.4) | 43 (5.9) | 31 (4.3) | 0.050 |
| Creatinine (μmol/L) | 89.72 (20.62) | 89.32 (17.81) | 92.46 (25.98) | 0.091 | 89.38 (18.79) | 89.34 (17.84) | 88.95 (21.98) | 0.014 |
| Creatinine clearance (ml/min) | 58.60 (14.59) | 60.44 (12.85) | 57.82 (16.28) | 0.121 | 58.82 (15.17) | 60.23 (11.28) | 59.08 (15.90) | 0.069 |
| SYNTAX score **^a^** |  |  |  | 0.600 |  |  |  | 0.045 |
| ≤22 | 135 (47.2) | 20 (15.5) | 76 (25.7) |  | 159 (22.7) | 150 (20.4) | 143 (20.0) |  |
| 23-32 | 103 (36.0) | 42 (32.6) | 108 (36.5) |  | 266 (37.8) | 284 (38.7) | 282 (39.4) |  |
| ≥ 33 | 47 (16.4) | 67 (51.9) | 111 (37.5) |  | 277 (39.5) | 300 (40.9) | 291 (40.6) |  |
| Medication upon discharge |  |  |  |  |  |  |  |  |
| Aspirin | 275 (96.2) | 119 (92.2) | 272 (91.9) | 0.120 | 667 (95.0) | 690 (94.0) | 669 (93.5) | 0.041 |
| Clopidogrel | 256 (89.5) | 11 (8.5) | 119 (40.2) | 1.587 | 592 (84.3) | 68 (9.3) | 261 (36.4) | 1.361 |
| Beta-blockers | 254 (84.6) | 103 (79.8) | 253 (85.5) | 0.105 | 609 (86.8) | 600 (81.7) | 613 (85.6) | 0.094 |
| Statins | 284 (99.3) | 128 (99.2) | 295 (99.7) | 0.026 | 698 (99.4) | 730 (99.5) | 710 (99.2) | 0.011 |
| Angiotensin converting enzyme inhibitors | 277 (96.9) | 126 (97.7) | 285 (96.3) | 0.044 | 682 (97.1) | 715 (97.4) | 693 (96.8) | 0.028 |
| Nitrates | 256 (89.5) | 117 (90.7) | 276 (93.2) | 0.089 | 626 (89.1) | 660 (89.9) | 660 (92.2) | 0.070 |
| Calcium Channel Blockers | 278 (97.2) | 126 (97.7) | 287(97.0) | 0.029 | 684 (97.4) | 716 (97.6) | 696 (97.2) | 0.021 |

ACS, Acute Coronary Syndrome. IPTW, inverse probability of treatment weighting. SMD, Standardized Mean Difference.

**^a^** Variables included in the propensity score.
