# Supplementary material for: Real-world long-term outcomes based on three therapeutic strategies in very old patients with three-vessel disease
Source: BMC Cardiovasc Disord. 2021 Jun 29;21:316. doi: 10.1186/s12872-021-02067-6 (PMC8243749; doi:10.1186/s12872-021-02067-6)
Supplement: Supplementary file 2 — Additional file 2: Table S2. Competing risks regression for cardiac death and other cardiovascular endpoints. [file 12872_2021_2067_MOESM2_ESM.docx]

**Table S2. Competing risks regression for cardiac death and other cardiovascular endpoints*.**

|  | PCI | CABG | | MT | |
| --- | --- | --- | --- | --- | --- |
|  |  | Hazard Ratio (95% Confidence Interval) | p | Hazard Ratio (95% Confidence Interval) | p |
| Cardiac Death | ref | 0.460 (0.224 – 0.947) | 0.035 | 1.723 (1.140 – 2.602) | 0.010 |
| Revascularization | ref | 0.287 (0.084 – 0.979) | 0.046 | 0.839 (0.434 – 1.620) | 0.600 |
| Myocardial Infarction | ref | 0.203 (0.048 – 0.875) | 0.032 | 0.957 (0.451 – 2.030) | 0.910 |
| Stroke | ref | 1.450 (0.722 – 2.910) | 0.300 | 0.694 (0.375 – 1.290) | 0.250 |

|  | MT | CABG | |
| --- | --- | --- | --- |
|  |  | Hazard Ratio (95% Confidence Interval) | p |
| Cardiac Death | ref | 0.267 (0.137 – 0.521) | <0.001 |
| Revascularization | ref | 0.341 (0.096 – 1.220) | 0.097 |
| Myocardial Infarction | ref | 0.212 (0.046 – 0.971) | 0.046 |
| Stroke | ref | 2.090 (1.040 – 4.220) | 0.040 |

MACCE = Major Adverse Cardiac and Cerebrovascular Events

*Competing risks regression with subdistribution hazard models for cardiac death and other cardiovascular endpoints, considering competing risks for non-cardiac death and all-cause death, respectively.
