# Supplementary material for: Real-world long-term outcomes based on three therapeutic strategies in very old patients with three-vessel disease
Source: BMC Cardiovasc Disord. 2021 Jun 29;21:316. doi: 10.1186/s12872-021-02067-6 (PMC8243749; doi:10.1186/s12872-021-02067-6)
Supplement: Supplementary file 3 — Additional file 3: Table S3. Inverse probability of treatment weighting (IPTW) regression analysis based on propensity score. [file 12872_2021_2067_MOESM3_ESM.docx]

**Table S3. Inverse probability of treatment weighting (IPTW) regression analysis based on propensity score*.**

|  | PCI | CABG | | MT | |
| --- | --- | --- | --- | --- | --- |
|  |  | Hazard Ratio (95% Confidence Interval) | p | Hazard Ratio (95% Confidence Interval) | p |
| All-cause Death | ref | 0.746 (0.481 – 1.158) | 0.191 | 1.287 (0.964 – 1.718) | 0.087 |
| Cardiac Death | ref | 0.439 (0.206 – 0.939) | 0.034 | 1.484 (0.983 – 2.243) | 0.061 |
| Revascularization | ref | 0.157 (0.040 – 0.606) | 0.007 | 0.859 (0.453 – 1.629) | 0.642 |
| Myocardial Infarction | ref | 0.106 (0.023 – 0.490) | 0.004 | 1.154 (0.561 – 2.374) | 0.697 |
| Stroke | ref | 1.787 (0.746 – 4.279) | 0.193 | 0.750 (0.398 – 1.415) | 0.375 |
| MACCE | ref | 0.690 (0.478 – 0.997) | 0.048 | 1.149 (0.902 – 1.463) | 0.260 |

|  | MT | CABG | |
| --- | --- | --- | --- |
|  |  | Hazard Ratio (95% Confidence Interval) | p |
| All-cause Death | ref | 0.634 (0.430 – 0.936) | 0.022 |
| Cardiac Death | ref | 0.301 (0.152 – 0.593) | <0.001 |
| Revascularization | ref | 0.329 (0.093 – 1.168) | 0.086 |
| Myocardial Infarction | ref | 0.178 (0.043 – 0.733) | 0.017 |
| Stroke | ref | 1.898 (0.921 – 3.913) | 0.083 |
| MACCE | ref | 0.710 (0.510 – 0.988) | 0.042 |

*The covariates included in propensity score model are as follows: age, sex, BMI，diabetes, hypertension, hyperlipidemia, previous MI, previous stroke, chronic kidney disease, peripheral artery disease, smoker, clinical presentation, left main disease, LVEF and SYNTAX score.
